# Informed consent at stake? Language barriers in medical interactions with immigrant anaesthetists: a conversation analytical study

**DOI:** 10.1186/s12913-019-4389-2

**Published:** 2019-08-23

**Authors:** Damaris Borowski, Uwe Koreik, Udo Ohm, Claudia Riemer, Niels Rahe-Meyer

**Affiliations:** 1 Center for Multilingualism and Language Acquisition (CEMES), University of Münster (Westfälische Wilhelms-Universität Münster), Robert-Koch-Str. 29, 48149 Münster, Germany; 2 Faculty of Linguistics and Literary Studies, Department of German as a Foreign Language, University of Bielefeld, Universitätsstr. 25, 33615 Bielefeld, Germany; 3 Department of Anaesthesiology and Intensive Care Medicine, Franziskus Hospital, Kiskerstraße 26, 33615 Bielefeld, Germany

**Keywords:** Doctor-patient-interaction, Pre-anaesthesia evaluation, Informed consent, Language barriers, Conversation analysis, Qualitative research

## Abstract

**Background:**

Language barriers in doctor-patient interactions are still an understudied phenomenon. This is particularly true concerning interactions with immigrant physicians who are learners of the patient’s language; there is a lack of research even though labour migration is increasing internationally. This conversation analytical study focusses on language errors in one specific type of doctor-patient interaction, namely pre-anaesthesia evaluations with immigrant anaesthetists.

**Methods:**

The study combines the research field of language acquisition with that of medical interaction. It is a qualitative study with an ethnomethodological framework which addresses the following research question: How do language errors, produced by immigrant anaesthetists, impact pre-anaesthesia evaluations? The primary data comes from naturally occurring pre-anaesthesia evaluations carried out by immigrant anaesthetists. The analysis method is a combination of conversation and error analysis.

**Results:**

The study shows that the anaesthetists produced a considerable number of unintelligible utterances, due to various language errors. Despite the lack of understanding, hardly any negotiation of meaning occurred and both sides (anaesthetists and patients) claimed to be satisfied.

**Conclusions:**

The findings appear to be contradictory. An explanation for this can be found in the effect of the roles and scripts that are given in pre-anaesthesia evaluations. Since no negotiation of meaning is initiated during the interactions, the anaesthetists’ insufficient language competence leads to a considerable impairment of informed consent, which is the main goal of the pre-anaesthesia evaluations. Based on these findings, the study reveals an urgent need for action regarding immigrant anaesthetists’ language skills.

## Background

Language barriers in medical interactions have been analysed in numerous studies: Moss et al. conducted studies with patients who speak English as their second language. Their analysis of the opening sequences showed that (due to the different cultural backgrounds) the different perceptions of the interactions’ course and content, as well as the linguistic barriers, increased the interactional effort needed. According to Moss et al., physicians have to endure a certain “interactional uncertainty”, in order to be able to handle patient heterogeneity appropriately [1]. Seelman and Suurmond conducted semi-standardized interviews on participatory decision-making with physicians and patients who spoke English as their second language. They concluded that linguistic and cultural barriers have a significant impact on decision-making and state, aptly, that “due to language difficulties, cultural differences, and bias, the process of information exchange may become highly distorted” [2]. Many more studies could be named (see e.g. [3–6]).

In summary, it can be stated that the earlier studies have reached the following key conclusions (see [7]): Language barriers can lead to a simplification or reduction of the interaction’s content. These barriers, as well as different perceptions of roles, scripts and procedures, complicate the interaction. It has been shown that linguistic and cultural differences have a medically and legally relevant impact on the interactions.

These findings relate to studies with patients who speak German or English as a second language. To date, interactions with physicians who speak English or German as a second language have received little attention in research, even though labour migration is an old phenomenon in both the United States and Europe: Migration flows to OECD countries, especially European countries, is steadily increasing [8]. In 2015 (i.e., the year in which the data for this study was collected), the German Medical Association reported a total of 189,622 physicians working in German hospitals [9]. Every sixth physician (30,595) was from abroad - three times the number compared to 2000. Most of the immigrant physicians come from other European countries (especially from Romania).

A variety of specific-purpose language training offerings (LSP) have been developed [10] in order to address the immediate and very specific language needs of immigrants in education, training or at work. The research interest for this study arose during teaching practice – i.e. German courses for immigrant physicians. In a documentation and critical reflection on one of these courses, Borowski named limited knowledge of the actual linguistic challenges faced by immigrant physicians at their workplace, as the crucial problem: The teaching materials and concepts for this type of course have considerable shortcomings due to this lack of research [11].

It must be stated, that while in clinical trials, medical interventions are strictly analysed and regulated, there are no control mechanisms for the extended area of everyday work, including doctor-patient-interactions: What do physicians and patients actually do in order to understand each other? Do language barriers occur in interactions with immigrant physicians? Do these language barriers have a negative effect on the interactions? Are the legal regulations and medical requirements fulfilled? Is it even possible to fulfil them?

Important questions, like these, are waiting to be answered. This study aims to take a first step in this direction, by addressing the following research question: How do immigrant anaesthetists’ language errors impact pre-anaesthesia evaluations?

## Methods

This study combines the research field of language acquisition with that of medical interaction. It does so by applying both conversation analysis and error analysis. In accordance with the conversation analytical approach, this study does not test a theory or develop categories by analysing data. Instead a detailed description and study of the documented interactions leads to new insights: “We will be using observations as a basis for theorizing. Thus we can start with things that are not currently imaginable, by showing that they happened” [12].

A detailed qualitative analysis of medical interaction requires that it be restricted to a specific type of interaction. As the above-stated research question shows, this study focusses on language barriers in interactions between anaesthetists and their patients in pre-anaesthesia evaluations. This type of interaction is particularly interesting because it has both a specific medical and legal relevance: The legal regulations and medical requirements must be fulfilled – this is especially evident when it comes to informed consent (see [13, 14]). For legal reasons, the physician must be sure that the patient has understood the information. The basis for informed consent is only created if the interaction partners confirm their mutual understanding (see [15]).

A qualitative analysis of naturally occurring pre-anaesthesia evaluations (this study’s primary data) was conducted in order to answer the research question (see [16] for the full study]. Many studies have already proven that the conversation analysis approach is valid and useful in the context of medical interactions [17–19]. For the specific objective of this study, conversation analysis was combined with a systematic analysis of language errors. This method is described below in the section on data analysis. In this way, it was possible to reveal the relationship between language barriers and restrictions to informed consent.

Secondary data (see below) was included in order to expand the understanding of the documented pre-anaesthesia evaluations in the given context. By using this ethnomethodological approach (see [20–22]), the study intersects with workplace studies (see [23]). The interviews and assessments included in the secondary data provide the interlocutors’ perspective. Therefore, it is possible to speak of methodological and theory triangulation (see [24] for detailed information).

All study procedures were approved by the legal departments of the University of Bielefeld and the Franziskus-Hospital, Bielefeld.

### Participants

Participation in the study was voluntary. All the participants signed a consent after having been fully informed about the study. All the participants had the right to withdraw their consent at any point during the study. Prior to data collection, the anaesthetists were informed that the focus of the study was on immigrant anaesthetists and the patients were informed that the purpose of the study was to improve pre-anaesthesia evaluations. After data collection, the patients were additionally informed of the specific focus on immigrant anaesthetists. Thus, the patients’ perception of the anaesthetists and their language proficiency, was not influenced during data collection. Three of the four anaesthetists approached agreed to participate in the study, as well as all the patients approached.

The participants in this study were:

- Three immigrant anaesthetists, i.e. anaesthetists who spoke German as a second language and had not studied medicine in Germany.
- Twelve patients. The patients were not selected – instead the first, third, fifth and seventh patient during the anaesthetists’ shifts were approached. Every second patient was left out, so that a short oral assessment could be conducted with the patients after their pre-anaesthesia evaluations without disrupting the anaesthetists’ work schedules.

### Data collection

Data was collected in November/December 2015. This study’s primary data are video recordings of naturally occurring, pre-anaesthesia evaluations: This means that the framework conditions were influenced as little as possible during data collection – most importantly, no changes were made to the roster, room or work routine. Only 3 of the 4 pre-anaesthesia evaluations recorded with each anaesthetist were analysed. In all cases, the first pre-anaesthesia evaluation was not included in the analysis; in this way, the camera’s influence on the anaesthetists’ conduct was reduced as they got used to the camera. It was found that the anaesthetists got into their normal work routine during the first videotaped, pre-anaesthesia evaluation (see [25] for detailed information). Pre-anaesthesia evaluations are always an exceptional situation for patients, during which they are preoccupied with their disease and the upcoming surgery (see [15]).

**Fig. 1:**
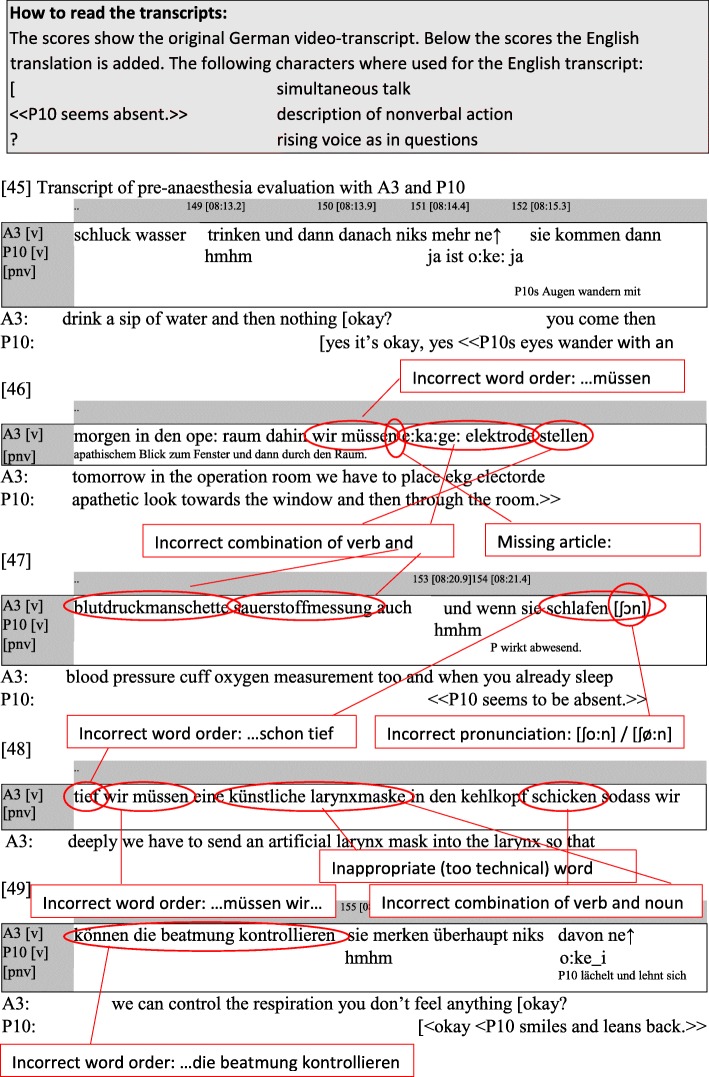
Transcript of pre-anaesthesia evaluation with A3 and P10

Primary data:

- Video recordings were made of 12 pre-anaesthesia evaluations between immigrant anaesthetists and their patients. The transcript in Fig. 1 gives an example of the transcripts made from these recordings.

Secondary data:

- Field notes: Written observations were made before, during and after the pre-anaesthesia evaluations, in order to document observations that were not captured by the cameras. The following is an example of the field notes which were written prior to the pre-anaesthesia evaluation between P10 and A3 (see Table 1 for patient and anaesthetist codes).
*Original: “A3 hat Schwierigkeiten mit dem Computerprogramm. Sie versucht sich anzumelden, gibt es dann aber auf. A3 erzählt mir, dass sie keine Einführung in die Programme bekommen habe.”*
  Translation: “A3 has difficulties with the computer program. She tries to log in, but then gives up. A3 tells me that she had not been given an introduction to the programs.”
- Documents: All documents used in connection with the pre-anaesthesia evaluations were copied and collected.
  - ○ 12 pre-anaesthesia evaluation information and medical history forms: Patients read the information given in these forms and fill in their medical history before the pre-anaesthesia evaluation. They take the form to the pre-anaesthesia evaluation and the anaesthetist refers to it during their interaction.
  - ○ 12 anaesthesia protocols: After each pre-anaesthesia evaluation, the anaesthetists write a short protocol for the anaesthetist who performs the anaesthesia on the day of surgery.
  - ○ 12 assessment forms: The anaesthetists completed a short additional form after each pre-anaesthesia evaluation, in order to document their immediate impression of the interactions for the study.
- Oral Assessments: Written notes were made of 12 short oral assessments with the patients after their pre-anaesthesia evaluations.
- Interviews: Audio recordings were made of 3 detailed interviews with the anaesthetists after the pre-anaesthesia evaluations.

**Table 1:** Duration of recorded pre-anaesthesia evaluations

A1	Minutes	A2	Minutes	A3	Minutes
P1	05:25	P5	13:45	P9	14:30
P2	13:08	P6	19:20	P10	06:15
P3	09:02	P7	27:53^a^	P11	5:03^a^
P4	06:51	P8	08:01	P12	05:53

A1-A3: anaesthetists

P1-P12: patients

^a^longest and shortest interaction

For more detailed information on data and data collection, please see [25].

### Data treatment and analysis

All data was strictly anonymized. The pre-anaesthesia evaluations, as well as the interviews with the anaesthetists, were transcribed in full using GAT2 conventions [26] and the EXMARaLDA transcription tool [27]. All the processed and anonymized data (i.e. transcripts, field notes and documents), are accessible online (see [28]).

The aim of investigating the impact of language barriers on the pre-anaesthesia evaluations, could be attained by combining two analyses:

1. An error analysis (see [29]) was performed on the transcribed pre-anaesthesia evaluations, in order to examine the anaesthetists’ language errors during the interactions. In the context of the study, language errors are defined as deviations from the norm for pre-anaesthesia evaluations, regarding any aspect of language i.e., phonetics/phonology, morphology, syntax, lexis, and pragmatics. In accordance with the interlanguage hypothesis, language errors in this study are understood as necessary steps in the process of language acquisition (see [30]).
2. A conversation analysis (see [31, 32]) was also performed which examined the course of the conversations (i.e., how understanding was established) and deviations (i.e., disturbances).

In contrast to Barkhuizen and Ellis’ example [33], the two procedures were initially carried out separately and the findings were subsequently related to each other. Furthermore, the pre-anaesthesia evaluations’ structure was described using Nowak’s system [34], so that the interactions could be compared with one another and with former studies, especially the study conducted by Klüber et al. on pre-anaesthesia evaluations with anaesthetists who speak German as their first language [15].

### Findings

As described in the section on method a detailed description and study of the documented interactions was conducted. In this publication it is not possible to present the transcripts of the interactions in full length including their detailed analyses. Instead key findings are summarized for this context. Please see [16] to view the complete study.

### Duration of the pre-anaesthesia evaluations

The following table shows the duration of the recorded pre-anaesthesia evaluations:

The combined length of the interactions analysed amounts to 2 h, 15 min and 6 s. The average length of the interactions was 11 min and 25 s.

### Anaesthetists

The first languages of the 3 participating anaesthetists were Romanian, Arabic, and English/Igbo. Igbo is one of the main languages of Nigeria and is rated among the Niger­Congo languages (see [35]). One anaesthetist grew up in Italy with Nigerian parents. Although she spoke English and Igbo with her parents and siblings, she spoke Italian with everyone else. For her, as well as for the anaesthetist who immigrated from Egypt, English had played a central role in her studies and previous professional experience. At the time the data was collected, the participating anaesthetists had already been working in Germany for 3 to 5 years. Two anaesthetists had graduated in an EU state, Romania and Italy, and one in Egypt.

Due to German regulations, the anaesthetists had to present a general language certificate documenting a B2 level in German (i.e. independent user) according to the Common European Framework of Reference for Languages (CEFR) [36]. In addition, a C1 level (i.e. proficient user) of German for medical purposes, was also required. The anaesthetists were only able to obtain a German work-permit by fulfilling these requirements.

### Patients

The pre-anaesthesia evaluation is usually the first and only encounter between the patient and the anaesthetist conducting the interaction. The patients have already received their diagnoses and are on the verge of surgical intervention. Disease types and patient groups vary from patient to patient, while the physician is required to adjust the interaction to suit the various situations, surgeries and patients. The patients who participated in this study all spoke German as their first language. In the videotaped pre-anaesthesia evaluations, the anaesthetists and patients only spoke German so that the patients’ further language skills were of no relevance. The age of the patients was between 4 and 82; one patient was a child, who was attended by its mother. The patients were about to undergo different types of surgeries (e.g. knee replacement surgery or transurethral bladder tumour resection) and anaesthetic procedures (e.g. general or plexus anaesthesia).

### Language errors which occurred

The error analysis revealed that all the anaesthetists made numerous basic language errors in their pre-anaesthesia evaluations. Basic language errors are understood as errors that are typically made by learners with A1 or A2 level language proficiency, according to the Common European Framework of Reference for Languages (CEFR) [36].

It was particularly noticeable that the pre-anaesthesia evaluations contained multiple sections, in which the language errors accumulated. The following example demonstrates this multiplication of errors. In it, the anaesthetist (A3) gives information about the procedure on the day of the surgery. The English translation attempts to convey the extract’s assumed meaning, disregarding the language errors.

*Original: sie kommen dann morgen in den ope: raum dahin wir müssen e:ka:ge: elektrode stellen blutdruckmanschette sauerstoffmessung auch und wenn sie schlafen schon [ʃɔn] tief wir müssen eine künstliche larynxmaske in den kehlkopf schicken sodass wir können die beatmung kontrollieren*

Translation: then tomorrow you will come to the operating theatre where we have to place ECG electrodes, as well as a blood pressure cuff and oxygen measurement and as soon as you are sleeping deeply, we have to put an artificial larynx mask into the larynx so that we can control the respiration

In this extract from a pre-anaesthesia evaluation, the anaesthetist makes numerous language errors: Incorrect word order, incorrect or inappropriate word choice, incorrect pronunciation, missing words, fragmentary phrases (see Fig. 1). In this example, the accumulated errors result in an incomprehensible utterance. At the very least, a medical layman could not understand the information which the anaesthetist is attempting to give.

Similar passages appeared in all the pre-anaesthesia evaluations analysed (see [37]). The analysis revealed that a considerable proportion of the information which the anaesthetists provided for the patients, was incomprehensible. Yet in spite of this lack of understanding, hardly any negotiation of meaning (e.g. querries) took place (see [37]).

**Fig. 2:**
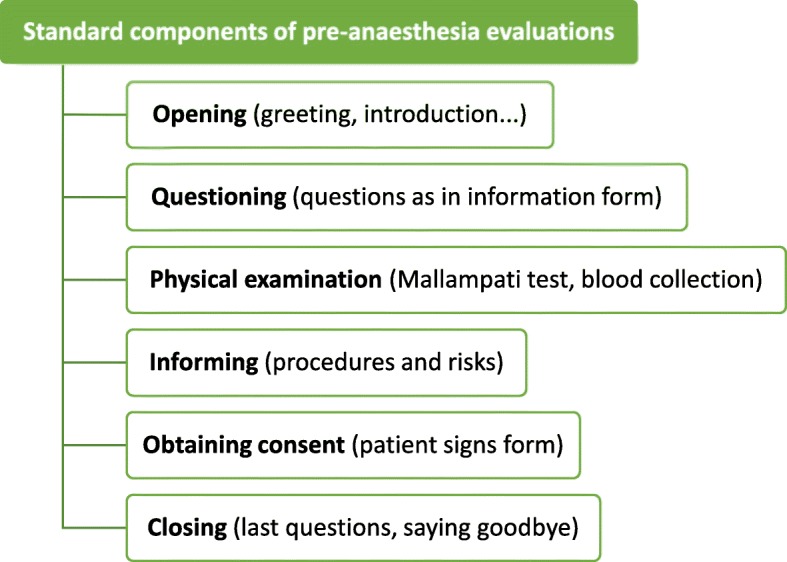
Standard components of pre-anaesthesia evaluations

It could be assumed that the standard elements (see Fig. 2) of a pre-anaesthesia evaluation do not represent a great challenge for the immigrant anaesthetists. Correspondingly, one of the anaesthetists explained in his interview that he had memorized all the standard elements of the pre-anaesthesia evaluations and merely reproduced them during the interactions. However, the analysis showed that language errors even accumulated in these regularly occurring elements. The example given above shows an accumulation of such errors in a standard element. In the pre-anaesthesia evaluations analysed, multiple language errors occurred in situations that were not exceptional but everyday routine (see [37] for variety examples).

The pre-anaesthesia evaluation extract quoted above, is an example of literal repetition of errors: A3 gave the same information, with the same wording and exactly the same errors during all her pre-anaesthesia evaluations. This leads to the conclusion that these errors have been repeated frequently over a long period. It can be assumed that they are now ingrained and can no longer be easily corrected. The same observation was made in respect of the other anaesthetists (see [37]).

### Patients’ lack of understanding

In the previous section it has been stated that due to the multitude of errors a considerable proportion of the anaesthetists’ information could not be understood by the patients. This observation emerged in the process of data analysis:

1. After the error analysis, extracts of the pre-anaesthesia evaluations were played to several native German speakers. (Only audio data was used here in order to preserve the anonymity of the participants). The native speakers were then asked to report what they had understood. It was found that a considerable amount of information was misunderstood or not understood at all. Even after listening to the extracts several times and additional reading of the transcripts, native speakers explained that they could not understand the given information.
2. The conversation analysis revealed that in the course of the pre-anaesthesia evaluations the patients’ lack of understanding only surfaced in sections where the anaesthetists asked questions. In these sections the patients’ missing or inappropriate answers revealed their lack of understanding. In a number of cases, the negotiation was aborted by the anaesthetists before understanding could be achieved. Since these sections extend over several transcript pages, they cannot be presented in this article. The transcripts (including these sections) are accessible online [28]. Borowski presents a detailed analysis of these sections [38].

### Anaesthetists’ and patients’ perspective

After every documented pre-anaesthesia evaluation, the patients were asked for an assessment of the interaction (see Table 2).

**Table 2:** Patients’ oral assessment

Patients’ oral assessment (*n* = 16)	
How satisfied are you with this pre-anaesthesia evaluation? 0 (not at all) – 10 (completely)	
Average:	8.7
Range:	7–10
How well did you understand the anaesthetists’ information/instructions? 0 (not at all) – 10 (without a problem)	
Average:	8.9
Range:	6–10
How well did the anaesthetist understand you? 0 (not at all) – 10 (without a problem)	
Average:	9.3
Range:	8–10

First, the patients were asked to rate their general satisfaction with the interaction using a numeric rating scale ranging from 0 (“not at all”) to 10 (“completely”). The patients’ assessments varied between 7 and 10, with an average satisfaction rating of 8.7. Next, they were asked how well (from 0 “not at all” to 10 “without a problem”) they could understand the anaesthetists’ information and instructions. The patients stated that they understood the anaesthetists well. The patients’ assessments varied between 6 and 10, with an average figure of 8.9. Moreover, the patients were asked to assess how well (from 0 “not at all” to 10 “without a problem”) they thought the anaesthetists had understood them. The patients’ assessments varied between 8 and 10, with an average figure of 9.3. Overall, it can be noted that the patients were satisfied with both the pre-anaesthesia evaluations and the anaesthetists’ language competence. They all felt that they were well informed and unanimously confirmed their consent. One patient was even persuaded by his anaesthetist to agree to an aesthetic procedure to which he had previously strongly objected (See [39] for a detailed presentation).

In their interviews, the immigrant anaesthetists judged their language competences overall positively. They felt that they could manage the pre-anaesthesia evaluations very well, were satisfied with their language skills and felt that the patients were also satisfied. This positive evaluation was confirmed by colleagues with German as their first language. “du hast das schnell gelernt ja wirklich” (you really learned quickly) or “du sprichst gut” (you talk well). The immigrant anaesthetists were convinced that they had never made any serious mistakes because of a lack of language or professional skills. They reported that no complaints had ever been filed because of their language skills, not even when they first started work (See [39] for a detailed presentation).

## Discussion

### Discussion of the main findings

The findings presented above (language errors which occurred, patients’ lack of understanding, participants’ perspective), seem to contradict each other: Even though the anaesthetists produced a considerable number of incomprehensible utterances, hardly any negotiation of meaning (e.g. questions posed by patients concerning the required information) took place and both sides claimed to be satisfied.

Based on these findings, it is possible to answer the research question – How do immigrant anaesthetists’ language errors impact pre-anaesthesia evaluations? – in two ways: The anaesthetists’ and patients’ survey leads to the conclusion that the immigrant anaesthetists’ language errors had no considerable impact on the pre-anaesthesia evaluations. However, the detailed qualitative analysis of the videotaped, pre-anaesthesia evaluations leads to an entirely different answer. As stated above (see methods), the basis of informed consent is that the patient understands the information given. However, the analysis revealed a multitude of unintelligible utterances due to language errors, even in standard elements of the pre-anaesthesia evaluations. Hence, the anaesthetists’ inadequate language competence leads to a considerable impairment of informed consent, which is the main goal of the pre-anaesthesia evaluations.

An explanation for this contradiction can be found in the effect of the roles and scripts that are given in pre-anaesthesia evaluations. Previous studies have already shown that the roles (i.e. anaesthetist and patient), and the script (i.e. standard elements) of the pre-anaesthesia evaluations help to bridge some of the communication problems (see [40]). This study shows that at the same time, these roles and scripts can also disguise communication problems: Both sides act their roles, regardless of whether understanding has been achieved. The roles and scripts explain why the patients did not indicate their lack of understanding (see section on patients’ lack of understanding): To do so would have involved interrupting the anaesthetist, which the patients probably did not consider part of their role. In addition, patients tend to blame their lack of understanding on themselves and consider failure to understand to be normal (see [37]). As a consequence, the anaesthetists were given the impression that informed consent had been obtained and no further negotiation was needed. Therefore, they assumed that there are no relevant language problems and saw no further need to continue improving their language skills.

The method used - a combination of conversation analysis and error analysis, proved fruitful and produced findings that would not have been revealed by a survey alone. This study shows that language errors are no longer discussed or reflected upon in the workplace, because mutual understanding is assumed. This inevitably leads to a standstill in language acquisition. The analysis of the interactions reveals that even the last possible control mechanism, i.e. the patients’ reaction, is missing. The result is an ongoing impairment of informed consent.

### Implications for language training

The study shows an urgent need for action regarding immigrant anaesthetists’ language skills. The following recommendations can be made for their language training:

- Anaesthetists not only need pre-vocational language training but continuous training after starting their jobs.
- Some of their training needs could be addressed in regular language courses.
- In addition, specific training for the medical purpose of anaesthesia is needed. This training should be offered at the workplace in order to address the actual language needed in this specific context and should include shadowing (see [41]) and professional feedback.

The data collected for this study could be utilized to create scenarios that could be used in specific language training for anaesthetists.

### Limitations of this study

As described in the method section, this study was restricted to 12 pre-anaesthesia evaluations with 3 anaesthetists and their patients. Working with relatively small numbers of participants is a limitation which accompanies every qualitative study. A subsequent quantitative study could be used to verify this study’s findings. As an alternative, further qualitative studies could be conducted on different types of interactions (e.g. interactions with colleagues) in different contexts (e.g. in the operating theatre) and different fields (e.g. geriatrics).

### Discussion of further findings

The duration of the interactions provided another unexpected result. It could be assumed that pre-anaesthesia evaluations with anaesthetists speaking a non-native language, would tend to be longer than other pre-anaesthesia evaluations because of the linguistic challenges. However, a comparison with the study conducted by Klüber et al. with anaesthetists who spoke German as their first language [15], refutes this assumption. The average length of the pre-anaesthesia evaluations recorded in that study was significantly longer: 17 m 15 s. Hence it can be concluded that pre-anaesthesia evaluations with immigrant anaesthetists do not usually take any longer than those performed by other anaesthetists. One explanation could be that language barriers lead to the interaction being reduced to basic problems (see [42]). This explanation could be verified in further studies by a systematic comparison between interactions with anaesthetists who are speaking a second language and anaesthetists who are speaking their first language.

**Fig. 3:**
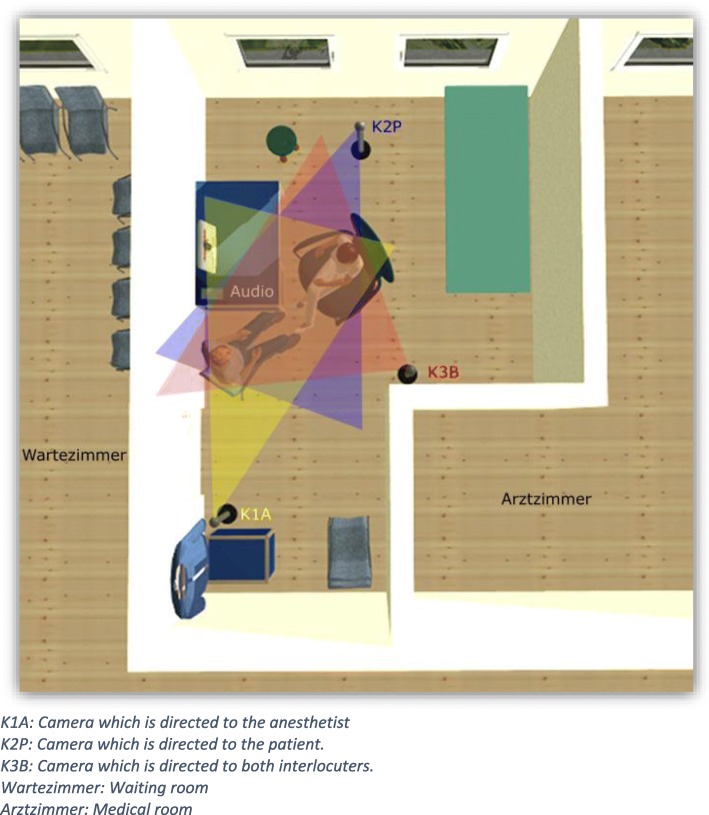
Camera positioning

For this study, the interactions were recorded using three cameras in such a fashion that all interactive resources used by the participants (see Fig. 3) were in focus. By this means, it was not only possible to record the verbal and other vocal resources (e.g., “er”, or coughing) but also multimodal resources, including gestures, eye movement, facial expression, body movement and posture, positioning and motion, as well as the use of objects (see [43]). This approach made it possible for example, to include the observations made during the physical examination.

Former conversation analytical studies on pre-anaesthesia evaluations (see [15, 34]) did not address the physical examination or regular disturbances by telephone conversations. Further studies should include these aspects of pre-anaesthesia evaluations.

## Conclusion

The study reveals an urgent need for action regarding immigrant anaesthetists’ language skills.

The analysis shows that these anaesthetists produce a considerable number of unintelligible utterances due to various language errors, even in standard elements. Despite the lack of understanding, hardly any negotiation of meaning occurs. Consequently the anaesthetists’ insufficient language competence leads to a considerable impairment of informed consent, which is the main goal of pre-anaesthesia evaluations.

It could also be shown that language errors are no longer discussed or reflected upon in the workplace, because mutual understanding is assumed. This inevitably leads to a standstill in language acquisition. The analysis of the interactions reveals that even the last possible control mechanism, i.e. the patients’ reaction, is missing. The result is an ongoing impairment of informed consent. Hence, informed consent – a matter of health, life and death – is indeed at stake.

## Data Availability

The datasets generated and analysed during this study are available (only in German) from the publisher Frank & Timme repository, http://www.frank-timme.de/fileadmin/docs/Borowski_Anhang.pdf

## Abbreviations

A: Anaesthetist
CEFR: Common European Framework of References for Languages
ECG: Electrocardiogram
GAT2: Gesprächsanalytisches Transkriptionssystem 2 (transcription convention)
LSP: Language for specific purpose
OECD: Organisation for Economic Co-operation and Development
P: Patient
